# Two-dimensional hydrogen-bonded polymers in the crystal structures of the ammonium salts of phen­oxy­acetic acid, (4-fluoro­phen­oxy)acetic acid and (4-chloro-2-methyl­phen­oxy)acetic acid

**DOI:** 10.1107/S160053681402488X

**Published:** 2014-11-19

**Authors:** Graham Smith

**Affiliations:** a Science and Engineering Faculty, Queensland University of Technology, GPO Box 2434, Brisbane, Queensland 4001, Australia

**Keywords:** crystal structure, phen­oxy­acetic acid salts, MCPA, herbicides, ammonium carboxyl­ates, hydrogen bonding

## Abstract

The structures of the ammonium salts of phen­oxy­acetic acid, NH_4_^+^·C_8_H_6_O_3_^−^, (I), (4-fluoro­phen­oxy)acetic acid, NH_4_^+^·C_8_H_5_FO_3_^−^, (II), and the herbicidally active (4-chloro-2-methyl­phen­oxy)acetic acid (MCPA), NH_4_^+^·C_9_H_8_ClO_3_^−^·0.5H_2_O, (III) have been determined. All have two-dimensional layered structures based on inter-species ammonium N—H⋯O hydrogen-bonding associations, which give core substructures consisting primarily of conjoined cyclic motifs. The crystals of (I) and (II) are isomorphous with the core comprising *R*_1_^2^(5), *R*_1_^2^(4) and centrosymmetric *R*_4_^2^(8) ring motifs, giving two-dimensional layers lying parallel to (100). In (III), the water mol­ecule of solvation lies on a crystallographic twofold rotation axis and bridges two carboxyl O atoms in an *R*_4_^4^(12) hydrogen-bonded motif, creating two *R*_4_^3^(10) rings, which together with a conjoined centrosymmetric *R*_4_^2^(8) ring incorporating both ammonium cations, generate two-dimensional layers lying parallel to (100). No π–π ring associations are present in any of the structures.

## Abstract 2

The crystal of the isomorphous anhydrous ammonium salts of phen­oxy­acetic acid and (4-fluoro­phen­oxy)acetic acid and that of the hemihydrate ammonium salt of 4-chloro-2-methyl­phen­oxy)acetic acid show two-dimensional layered structures based on conjoined cyclic hydrogen-bonded motifs.

## Chemical context   

The crystal structures of the ammonium salts of carb­oxy­lic acids are, despite their simple formulae, characterized by the presence of a complex array of hydrogen-bonding inter­actions. From a study of the packing motifs of the these ammonium carboxyl­ate salts from examples in the Cambridge Structural Database (Groom & Allen, 2014 ▶), Odendal *et al.* (2010 ▶) found that two-dimensional hydrogen-bonded nets, ladders or cubane-type structures could be predicted on the basis of the size and conformation of the anions. These structures are often stabilized by π–π aromatic ring inter­actions. With the benzoic acid analogues, two-dimensional sheet structures are common with inter­actions involving the ammonium cations and the carboxyl­ate anions in N—H⋯O hydrogen bonding, forming core layer structures, with the aromatic rings occupying the inter­stitial cell regions, *e.g.* with benzoic acid (Odendal *et al.*, 2010 ▶), 3-nitro­benzoic acid (Eppel & Bernstein, 2009 ▶) and 2,4-di­chloro­benzoic acid (Smith, 2014 ▶). Three-dimensional structures are usually only formed when inter­active substituent groups are present on the benzoate rings, inter­linking the layers *e.g.* with 3,5-di­nitro­benzoic acid (Smith, 2014 ▶). The presence of water mol­ecules of solvation may also produce a similar effect, although these are usually confined to the primary cation–anion layers.

With the phen­oxy­acetic acid analogues, which comprise a number of herbicidally active commercial herbicides (Zumdahl, 2010 ▶), this should also be the case. In the only reported structure of an ammonium salt of a phen­oxy­acetic acid [with the commercially important herbicide, the 2,4-di­chloro-substituted analogue (2,4-D) (a hemihydrate) (Liu *et al.*, 2009 ▶)], the expected two-dimensional layered structure is found. Herein are reported the preparation and structures of the anhydrous ammonium salts of the parent phen­oxy­acetic acid, NH_4_^+^·C_8_H_6_O_3_^−^ (I) and (4-fluoro­phen­oxy)acetic acid, NH_4_^+^·C_8_H_5_FO_3_^−^ (II) and the hemihydrate salt of the herbicidally active (4-chloro-2-methyl­phen­oxy)acetic acid (MCPA), NH_4_^+^·C_9_H_8_ClO_3_^−^·0.5H_2_O (III). The structure of a hydrated chloro­methyl­ammonium salt of MCPA is known (Pernak *et al.*, 2011 ▶).
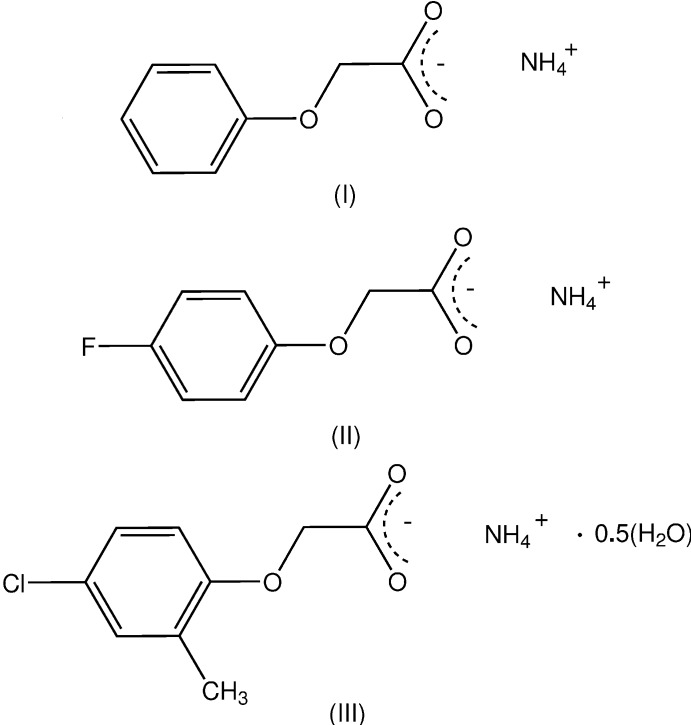


## Structural commentary   

**Figure 1:**
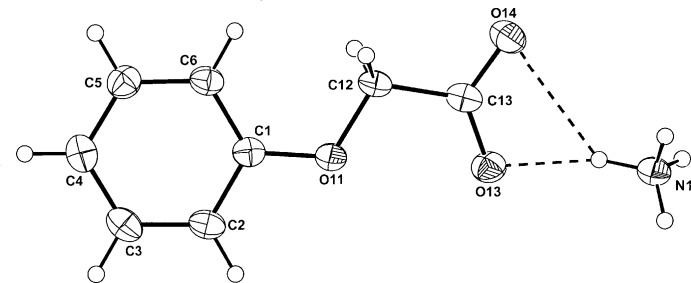
Mol­ecular conformation and atom labelling for (I), with inter-species hydrogen bonds shown as a dashed lines (see Table 1 ▶ for details). Non-H atoms are shown as 40% probability displacement ellipsoids.

**Figure 3:**
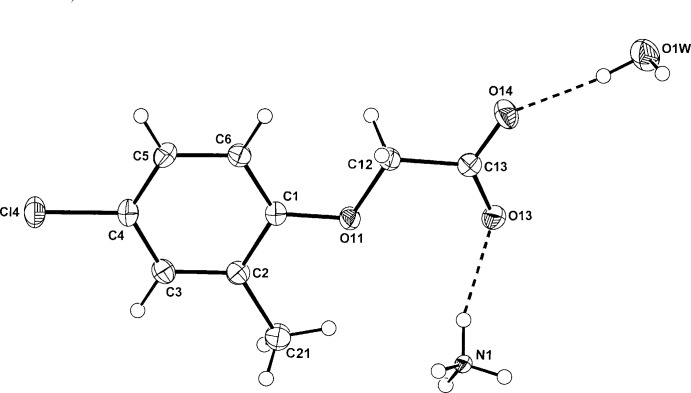
Mol­ecular conformation and atom labelling for (III), with inter-species hydrogen bonds shown as dashed lines (see Table 3 ▶ for details). Non-H atoms are shown as 40% probability displacement ellipsoids.

In the structures of the isomorphous ammonium phen­oxy­acetate (I) and (4-fluoro­phen­oxy)acetate (II) (Figs. 1 ▶ and 2 ▶, respectively), the anionic species are essentially planar; the comparative defining torsion angles in the phen­oxy­acetate side chain (C2—C1—O11—C12, C1—O11—C12—C13 and O11—C12—C13—O14) are 178.93 (19), −177.48 (18) and −173.58 (18)°, respectively, for (I) and −179.05 (18), −178.98 (17) and −174.13 (17)°, respectively, for (II). This planarity is also found in the MCPA anion in (III) (Fig. 3 ▶) where the corresponding torsion angles are −179.13 (15), −173.34 (14) and −178.71 (15)° and is also the case with the parent acids [for (I): Kennard *et al.* (1982 ▶), for (II): Smith *et al.* (1992 ▶) and for (III): Smith & Kennard (1981 ▶); Sieron *et al.* (2011 ▶)]. In (III), the water mol­ecule of solvation lies on a crystallographic twofold rotation axis.

## Supra­molecular features   

**Figure 4:**
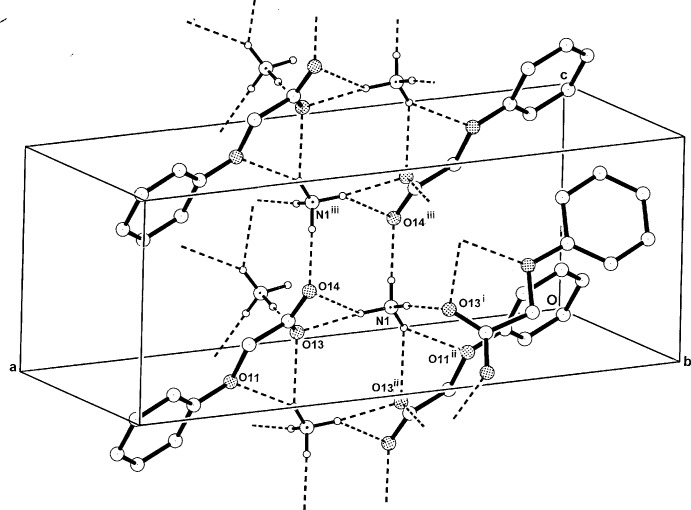
The two-dimensional hydrogen-bonded network structure of (I), which is equivalent to that of the isomorphous compound (II). Hydrogen bonds are shown as dashed lines and non-associative H-atoms have been omitted [for symmetry codes see Tables 1 ▶ and 2 ▶].

In the crystals of (I) and (II), two H atoms of the ammonium group give cyclic asymmetric three-centre (bifurcated) N—H⋯(O,O) hydrogen-bonding inter­actions with the anion (Tables 1 ▶ and 2 ▶, respectively). One of these is with two O-atom acceptors of the carboxyl group (O13, O14) [graph set 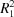
(4)], the other is with the carboxyl and phen­oxy O-atom acceptors (O13^ii^, O11^ii^) of an inversion-related anion [graph set 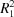
(5)]. These, together with a third N1—H13⋯O13^ii^ hydrogen bond, give a cyclic 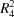
(8) ring motif, forming a series of conjoined rings which extend the structures along *c*. The other H atom gives structure extension through an N—H⋯O hydrogen bond to a carboxyl O atom (O14^iii^), forming a two-dimensional sheet-like structure which lies parallel to (100). Present in the crystal are short inversion-related inter­molecular F4⋯F4^iv^ contacts of 2.793 (2) Å [symmetry code: (iv) −*x* + 2, −*y* + 1, −*z* − 1]. The crystal packing and hydrogen-bonding in (I) is identical to that in isostructural (II), as shown in Fig. 4 ▶.

**Figure 5:**
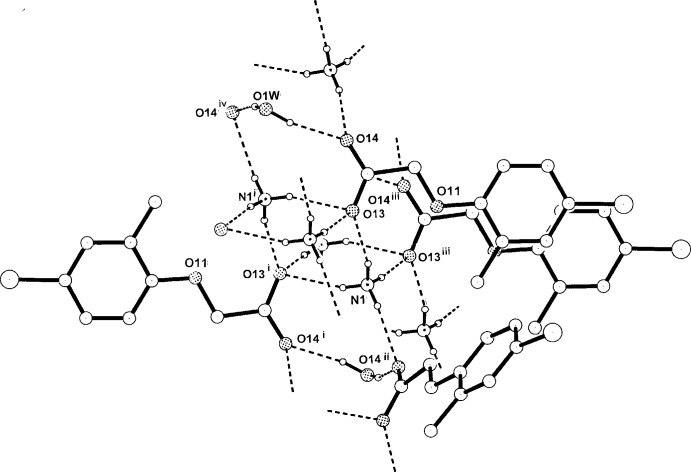
A partial extension of the basic cation–anion hydrogen-bonding associations in the structure of (III), showing conjoined cyclic 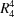
(12), 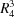
(10) and 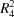
(8) ring motifs. [Symmetry code: (iv) −*x* + 1, *y*, −*z* + 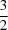. For other codes, see Table 3 ▶].

**Figure 6:**
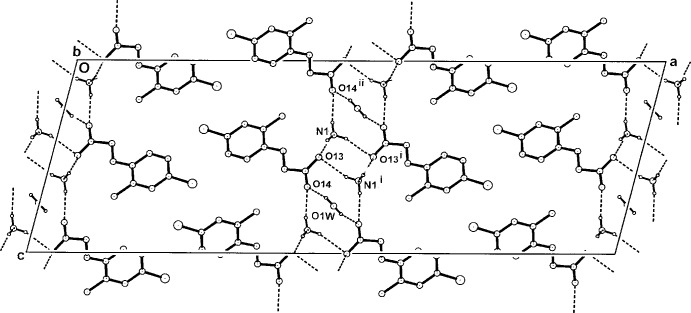
The two-dimensional hydrogen-bonded network structure of (III) in the unit cell, viewed along *b*.

In the crystal of (III), centrosymmetric inter-ion 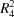
(8) rings are formed between two ammonium cations and two O13 carboxyl O-atom acceptors and are bridged by a third ammonium H donor through O13^iii^, extending the structure down *b* (Table 3 ▶ and Fig. 5 ▶). The fourth H atom gives extension along *a* through N1—H12⋯O14^ii^ forming an enlarged conjoined 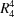
(12) ring, which is bridged by the water mol­ecule of solvation lying on the twofold rotation axis, through O1*W*—H11*W*⋯O14 hydrogen bonds. This link effectively generates two separate 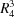
(10) ring motifs, extending the structure along *a* and giving the overall two-dimensional layers lying parallel to (100) (Fig. 6 ▶). In (III), no three-centre 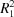
(4) or 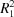
(5) motifs to carboxyl (*O*,*O*′) or carboxyl-phen­oxy (*O*,*O* ^1^) acceptors such as are present in (I) and (II) are found. The structure of (III) is essentially isostructural with that of ammonium (2,4-di­chloro­phen­oxy)acetate hemihydrate (Liu *et al.*, 2009 ▶), with isomorphous crystals [*a* = 37.338 (8), *b* = 4.388 (9), *c* = 12.900 (3) Å, β = 103.82 (3)°, *V* = 2074.7 (8) Å^3^, *Z* = 8, space group *C*2/*c*].

No π–π inter­actions are found in any of the structures reported here [minimum ring centroid separation = 4.8849 (16) (I), 4.8919 (15) (II) and 4.456 (5) Å (III) (the *b* unit-cell parameter)].

## Synthesis and crystallization   

The title compounds were prepared by the addition of excess 5 *M* aqueous ammonia solution to 1 mmol of either phen­oxy­acetic acid [150 mg for (I)], (4-fluoro­phen­oxy)acetic acid [170 mg for (II)] or (4-chloro-2-methyl­phen­oxy)acetic acid [200 mg for (III)] in 10 mL of 10% ethanol–water. Room-temperature evaporation of the solvent gave colourless plate-like crystals of (I), (II) and (III) from which specimens were cleaved for the X-ray analyses.

## Refinement details   

Crystal data, data collection and structure refinement details are summarized in Table 4 ▶. Hydrogen atoms potentially involved in hydrogen-bonding inter­actions were located in difference Fourier maps but were subsequently included in the refinements with positional parameters fixed and with *U*_iso_(H) = 1.2*U*_eq_(N) or = 1.5*U*_eq_(O). Other H atoms were included at calculated positions [C—H(aromatic) = 0.95, C—H(methyl­ene) = 0.98, C—H(meth­yl) = 0.97 Å] and also treated as riding, with *U*_iso_(H) = 1.5*U*_eq_(C) for methyl H atoms and = 1.2*U*_eq_(C) for other H atoms. In (III), the methyl group was found to be rotationally disordered, with the H atoms distributed over six equivalent half-sites, and was treated accordingly.

## Supplementary Material

Crystal structure: contains datablock(s) global, I, II, III. DOI: 10.1107/S160053681402488X/su5018sup1.cif

Structure factors: contains datablock(s) I. DOI: 10.1107/S160053681402488X/su5018Isup2.hkl

Structure factors: contains datablock(s) II. DOI: 10.1107/S160053681402488X/su5018IIsup3.hkl

Structure factors: contains datablock(s) III. DOI: 10.1107/S160053681402488X/su5018IIIsup4.hkl

Supporting information file. DOI: 10.1107/S160053681402488X/su5018Isup5.cml

Supporting information file. DOI: 10.1107/S160053681402488X/su5018IIsup6.cml

Supporting information file. DOI: 10.1107/S160053681402488X/su5018IIIsup7.cml

CCDC references: 1033945, 1033946, 1033947

Additional supporting information:  crystallographic information; 3D view; checkCIF report

## Figures and Tables

**Figure 2:**
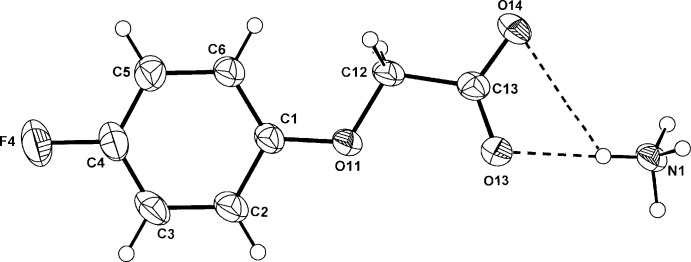
Mol­ecular conformation and atom labelling for (II), with inter-species hydrogen bonds shown as dashed lines (see Table 2 ▶ for details). Non-H atoms are shown as 40% probability displacement ellipsoids.

**Table 1:** Hydrogen-bond geometry (, ) for (I)

*D*H*A*	*D*H	H*A*	*D* *A*	*D*H*A*
N1H11O13	0.96	1.92	2.849(3)	163
N1H11O14	0.96	2.55	3.330(3)	138
N1H12O13^i^	0.85	2.03	2.867(3)	172
N1H13O11^ii^	0.90	2.39	3.202(3)	150
N1H13O13^ii^	0.90	2.15	2.869(3)	136
N1H14O14^iii^	0.84	1.95	2.788(3)	178

Symmetry codes: (i) 
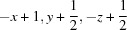
; (ii) 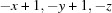; (iii) 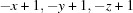.

**Table 2:** Hydrogen-bond geometry (, ) for (II)

*D*H*A*	*D*H	H*A*	*D* *A*	*D*H*A*
N1H11O13	0.90	1.95	2.847(2)	177
N1H11O14	0.90	2.55	3.347(2)	135
N1H12O13^i^	0.97	1.88	2.847(3)	173
N1H13O11^ii^	0.96	2.36	3.172(2)	142
N1H13O13^ii^	0.96	2.13	2.892(2)	135
N1H14O14^iii^	0.89	1.91	2.793(2)	173

Symmetry codes: (i) 
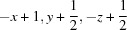
; (ii) 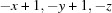; (iii) 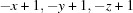.

**Table 3:** Hydrogen-bond geometry (, ) for (III)

*D*H*A*	*D*H	H*A*	*D* *A*	*D*H*A*
N1H11O13^i^	0.82	2.21	2.998(4)	161
N1H12O14^ii^	0.82	2.09	2.886(4)	166
N1H13O13^iii^	0.84	2.04	2.877(4)	173
N1H14O13	0.82	2.00	2.798(4)	163
O1*W*H11*W*O14	0.88	1.95	2.809(4)	165

Symmetry codes: (i) 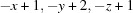; (ii) 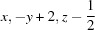; (iii) 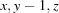.

**Table 4:** Experimental details

	(I)	(II)	(III)
Crystal data			
Chemical formula	NH_4_^+^C_8_H_7_O_3_	NH_4_^+^C_8_H_6_FO_3_	NH_4_^+^C_9_H_8_ClNO_3_ 0.5H_2_O
*M*_r_	169.17	187.17	226.65
Crystal system, space group	Monoclinic, *P*2_1_/*c*	Monoclinic, *P*2_1_/*c*	Monoclinic, *C*2/*c*
Temperature (K)	200	200	200
*a*, *b*, *c* ()	17.824(2), 7.1453(6), 6.7243(7)	18.386(2), 7.1223(6), 6.7609(6)	38.0396(9), 4.4560(8), 12.944(5)
()	90.321(9)	93.399(8)	104.575(5)
*V* (^3^)	856.38(15)	883.79(14)	2123.5(9)
*Z*	4	4	8
Radiation type	Mo *K*	Mo *K*	Mo *K*
(mm^1^)	0.10	0.12	0.35
Crystal size (mm)	0.35 0.25 0.10	0.26 0.20 0.05	0.35 0.35 0.10

Data collection			
Diffractometer	Oxford Diffraction Gemini-S CCD detector	Oxford Diffraction Gemini-S CCD detector	Oxford Diffraction Gemini-S CCD detector
Absorption correction	Multi-scan (*CrysAlis PRO*; Agilent, 2013 ▶)	Multi-scan (*CrysAlis PRO*; Agilent, 2013 ▶)	Multi-scan (*CrysAlis PRO*; Agilent, 2013 ▶)
*T*_min_, *T*_max_	0.920, 0.980	0.960, 0.980	0.913, 0.980
No. of measured, independent and observed [*I* > 2(*I*)] reflections	5450, 1686, 1218	5619, 1738, 1304	6215, 2087, 1771
*R*_int_	0.052	0.033	0.030
(sin /)_max_ (^1^)	0.617	0.617	0.617

Refinement			
*R*[*F* ^2^ > 2(*F* ^2^)], *wR*(*F* ^2^), *S*	0.063, 0.163, 1.10	0.053, 0.116, 1.10	0.036, 0.091, 1.03
No. of reflections	1686	1738	2087
No. of parameters	109	118	132
H-atom treatment	H-atom parameters constrained	H-atom parameters constrained	H-atom parameters constrained
_max_, _min_ (e ^3^)	0.29, 0.24	0.16, 0.22	0.32, 0.28

Computer programs: *CrysAlis PRO* (Agilent, 2013 ▶), *SIR92* (Altomare *et al.*, 1993 ▶), *SHELXS97* and *SHELXL97* (Sheldrick, 2008 ▶) within *WinGX* (Farrugia, 2012 ▶) and *PLATON* (Spek, 2009 ▶).

## supplementary crystallographic information

### Crystal data

**Table:**

NH_4_^+^·C_9_H_8_ClNO_3_^−^·0.5H_2_O	*F*(000) = 952
*M**_r_* = 226.65	*D*_x_ = 1.418 Mg m^−^^3^
Monoclinic, *C*2/*c*	Mo *K*α radiation, λ = 0.71073 Å
Hall symbol: -C 2yc	Cell parameters from 1819 reflections
*a* = 38.0396 (9) Å	θ = 4.4–28.1°
*b* = 4.4560 (8) Å	µ = 0.35 mm^−^^1^
*c* = 12.944 (5) Å	*T* = 200 K
β = 104.575 (5)°	Plate, colourless
*V* = 2123.5 (9) Å^3^	0.35 × 0.35 × 0.10 mm
*Z* = 8	

### Data collection

**Table:**

Oxford Diffraction Gemini-S CCD-detector diffractometer	2087 independent reflections
Radiation source: Enhance (Mo) X-ray source	1771 reflections with *I* > 2σ(*I*)
Graphite monochromator	*R*_int_ = 0.030
Detector resolution: 16.077 pixels mm^-1^	θ_max_ = 26.0°, θ_min_ = 3.2°
ω scans	*h* = −46→46
Absorption correction: multi-scan (*CrysAlis PRO*; Agilent, 2013)	*k* = −5→5
*T*_min_ = 0.913, *T*_max_ = 0.980	*l* = −15→15
6215 measured reflections	

### Refinement

**Table:**

Refinement on *F*^2^	Primary atom site location: structure-invariant direct methods
Least-squares matrix: full	Secondary atom site location: difference Fourier map
*R*[*F*^2^ > 2σ(*F*^2^)] = 0.036	Hydrogen site location: inferred from neighbouring sites
*wR*(*F*^2^) = 0.091	H-atom parameters constrained
*S* = 1.03	*w* = 1/[σ^2^(*F*_o_^2^) + (0.0409*P*)^2^ + 1.4504*P*] where *P* = (*F*_o_^2^ + 2*F*_c_^2^)/3
2087 reflections	(Δ/σ)_max_ < 0.001
132 parameters	Δρ_max_ = 0.32 e Å^−^^3^
0 restraints	Δρ_min_ = −0.28 e Å^−^^3^

### Special details

**Table:**

Geometry. Bond distances, angles *etc*. have been calculated using the rounded fractional coordinates. All su's are estimated from the variances of the (full) variance-covariance matrix. The cell e.s.d.'s are taken into account in the estimation of distances, angles and torsion angles
Refinement. Refinement of *F*^2^ against ALL reflections. The weighted *R*-factor *wR* and goodness of fit *S* are based on *F*^2^, conventional *R*-factors *R* are based on *F*, with *F* set to zero for negative *F*^2^. The threshold expression of *F*^2^ > σ(*F*^2^) is used only for calculating *R*-factors(gt) *etc*. and is not relevant to the choice of reflections for refinement. *R*-factors based on *F*^2^ are statistically about twice as large as those based on *F*, and *R*- factors based on ALL data will be even larger.

### Fractional atomic coordinates and isotropic or equivalent isotropic displacement parameters (Å^2^)

**Table:**

	*x*	*y*	*z*	*U*_iso_*/*U*_eq_	Occ. (<1)
Cl4	0.24818 (1)	0.32330 (12)	0.36021 (4)	0.0372 (2)	
O11	0.39197 (3)	0.8996 (3)	0.46961 (9)	0.0262 (4)	
O13	0.45425 (3)	1.2161 (3)	0.49906 (10)	0.0296 (4)	
O14	0.44789 (4)	1.4021 (3)	0.65332 (10)	0.0339 (4)	
C1	0.35808 (5)	0.7686 (4)	0.44981 (13)	0.0224 (5)	
C2	0.34876 (5)	0.5869 (4)	0.35836 (13)	0.0246 (5)	
C3	0.31491 (5)	0.4496 (4)	0.33324 (14)	0.0269 (5)	
C4	0.29091 (5)	0.4918 (4)	0.39679 (14)	0.0264 (5)	
C5	0.30027 (5)	0.6675 (4)	0.48684 (14)	0.0275 (6)	
C6	0.33390 (5)	0.8070 (4)	0.51321 (14)	0.0258 (5)	
C12	0.40205 (5)	1.0812 (4)	0.56298 (14)	0.0242 (5)	
C13	0.43762 (5)	1.2444 (4)	0.57156 (14)	0.0234 (5)	
C21	0.37517 (5)	0.5430 (5)	0.29002 (15)	0.0370 (6)	
O1W	0.50000	1.8306 (4)	0.75000	0.0464 (7)	
N1	0.46781 (3)	0.7274 (3)	0.37900 (10)	0.0156 (4)	
H3	0.30820	0.32800	0.27310	0.0320*	
H5	0.28420	0.69260	0.52960	0.0330*	
H6	0.34030	0.92710	0.57380	0.0310*	
H121	0.40420	0.95550	0.62550	0.0290*	
H122	0.38300	1.22720	0.56190	0.0290*	
H211	0.39700	0.65450	0.31980	0.0550*	0.500
H212	0.36440	0.61300	0.21890	0.0550*	0.500
H213	0.38090	0.33380	0.28790	0.0550*	0.500
H214	0.36460	0.41300	0.23130	0.0550*	0.500
H215	0.39710	0.45450	0.33210	0.0550*	0.500
H216	0.38060	0.73370	0.26320	0.0550*	0.500
H11W	0.48390	1.71260	0.70860	0.0700*	
H11	0.49010	0.73870	0.39810	0.0190*	
H12	0.45970	0.71470	0.31450	0.0190*	
H13	0.46250	0.57360	0.40950	0.0190*	
H14	0.45930	0.86670	0.40680	0.0190*	

### Atomic displacement parameters (Å^2^)

**Table:**

	*U*^11^	*U*^22^	*U*^33^	*U*^12^	*U*^13^	*U*^23^
Cl4	0.0264 (3)	0.0466 (3)	0.0378 (3)	−0.0121 (2)	0.0064 (2)	−0.0044 (2)
O11	0.0233 (7)	0.0335 (7)	0.0230 (6)	−0.0071 (5)	0.0083 (5)	−0.0075 (5)
O13	0.0293 (7)	0.0279 (6)	0.0355 (7)	−0.0036 (6)	0.0155 (6)	−0.0015 (6)
O14	0.0340 (8)	0.0378 (7)	0.0268 (7)	−0.0094 (6)	0.0020 (6)	−0.0054 (6)
C1	0.0215 (9)	0.0222 (8)	0.0224 (9)	−0.0010 (7)	0.0037 (7)	0.0028 (7)
C2	0.0253 (9)	0.0277 (9)	0.0198 (8)	0.0005 (7)	0.0041 (7)	0.0002 (7)
C3	0.0278 (10)	0.0287 (9)	0.0223 (9)	−0.0021 (8)	0.0027 (8)	−0.0023 (8)
C4	0.0217 (9)	0.0277 (9)	0.0277 (9)	−0.0031 (7)	0.0023 (8)	0.0037 (8)
C5	0.0230 (10)	0.0332 (10)	0.0282 (9)	0.0003 (8)	0.0099 (8)	0.0017 (8)
C6	0.0259 (10)	0.0281 (9)	0.0234 (9)	−0.0011 (8)	0.0062 (7)	−0.0022 (8)
C12	0.0244 (9)	0.0276 (9)	0.0208 (8)	−0.0026 (7)	0.0059 (7)	−0.0033 (7)
C13	0.0247 (9)	0.0215 (8)	0.0232 (9)	0.0029 (7)	0.0045 (7)	0.0042 (7)
C21	0.0331 (11)	0.0520 (12)	0.0278 (10)	−0.0083 (10)	0.0114 (9)	−0.0126 (9)
O1W	0.0421 (13)	0.0259 (10)	0.0667 (15)	0.0000	0.0053 (11)	0.0000
N1	0.0141 (7)	0.0159 (6)	0.0179 (6)	−0.0006 (5)	0.0060 (5)	−0.0004 (5)

### Geometric parameters (Å, º)

**Table:**

Cl4—C4	1.744 (3)	C3—C4	1.387 (3)
O11—C1	1.379 (3)	C4—C5	1.375 (3)
O11—C12	1.425 (3)	C5—C6	1.386 (3)
O13—C13	1.263 (3)	C12—C13	1.515 (3)
O14—C13	1.248 (3)	C3—H3	0.9300
O1W—H11W	0.8800	C5—H5	0.9300
O1W—H11W^i^	0.8800	C6—H6	0.9300
N1—H12	0.8200	C12—H121	0.9700
N1—H11	0.8200	C12—H122	0.9700
N1—H13	0.8400	C21—H216	0.9600
N1—H14	0.8200	C21—H211	0.9600
C1—C2	1.404 (3)	C21—H212	0.9600
C1—C6	1.389 (3)	C21—H213	0.9600
C2—C21	1.509 (3)	C21—H214	0.9600
C2—C3	1.388 (3)	C21—H215	0.9600
			
C1—O11—C12	115.95 (13)	C2—C3—H3	120.00
H11W—O1W—H11W^i^	107.00	C4—C3—H3	120.00
H12—N1—H14	114.00	C4—C5—H5	120.00
H13—N1—H14	104.00	C6—C5—H5	120.00
H11—N1—H12	114.00	C5—C6—H6	120.00
H11—N1—H13	105.00	C1—C6—H6	120.00
H11—N1—H14	108.00	C13—C12—H122	109.00
H12—N1—H13	111.00	C13—C12—H121	109.00
O11—C1—C2	115.26 (16)	O11—C12—H121	109.00
O11—C1—C6	124.41 (15)	O11—C12—H122	109.00
C2—C1—C6	120.33 (17)	H121—C12—H122	108.00
C1—C2—C21	120.32 (17)	C2—C21—H211	109.00
C1—C2—C3	118.30 (17)	C2—C21—H212	109.00
C3—C2—C21	121.37 (16)	C2—C21—H213	109.00
C2—C3—C4	120.77 (16)	C2—C21—H214	110.00
C3—C4—C5	120.76 (18)	C2—C21—H215	109.00
Cl4—C4—C3	119.22 (14)	C2—C21—H216	109.00
Cl4—C4—C5	120.01 (15)	H214—C21—H215	109.00
C4—C5—C6	119.37 (17)	H214—C21—H216	109.00
C1—C6—C5	120.46 (16)	H215—C21—H216	110.00
O11—C12—C13	112.31 (15)	H211—C21—H212	109.00
O13—C13—O14	125.29 (18)	H211—C21—H213	110.00
O13—C13—C12	120.17 (16)	H212—C21—H213	109.00
O14—C13—C12	114.55 (16)		
			
C12—O11—C1—C2	−179.13 (15)	C1—C2—C3—C4	0.2 (3)
C12—O11—C1—C6	1.0 (2)	C21—C2—C3—C4	179.98 (17)
C1—O11—C12—C13	−173.34 (14)	C2—C3—C4—Cl4	178.25 (14)
O11—C1—C2—C3	−179.57 (15)	C2—C3—C4—C5	−0.8 (3)
O11—C1—C2—C21	0.6 (2)	Cl4—C4—C5—C6	−178.13 (14)
C6—C1—C2—C3	0.3 (3)	C3—C4—C5—C6	0.9 (3)
C6—C1—C2—C21	−179.46 (17)	C4—C5—C6—C1	−0.4 (3)
O11—C1—C6—C5	179.67 (16)	O11—C12—C13—O13	1.7 (2)
C2—C1—C6—C5	−0.2 (3)	O11—C12—C13—O14	−178.71 (15)

Symmetry code: (i) −*x*+1, *y*, −*z*+3/2.

### Hydrogen-bond geometry (Å, º)

**Table:**

*D*—H···*A*	*D*—H	H···*A*	*D*···*A*	*D*—H···*A*
N1—H11···O13^ii^	0.82	2.21	2.998 (4)	161
N1—H12···O14^iii^	0.82	2.09	2.886 (4)	166
N1—H13···O13^iv^	0.84	2.04	2.877 (4)	173
N1—H14···O13	0.82	2.00	2.798 (4)	163
O1*W*—H11*W*···O14	0.88	1.95	2.809 (4)	165

Symmetry codes: (ii) −*x*+1, −*y*+2, −*z*+1; (iii) *x*, −*y*+2, *z*−1/2; (iv) *x*, *y*−1, *z*.

## References

[bb1] Agilent (2013). *CrysAlis PRO*. Agilent Technologies Ltd, Yarnton, England.

[bb2] Altomare, A., Cascarano, G., Giacovazzo, C. & Guagliardi, A. (1993). *J. Appl. Cryst.* **26**, 343–350.

[bb3] Eppel, S. & Bernstein, J. (2009). *Cryst. Growth Des.* **9**, 1683–1691.

[bb4] Farrugia, L. J. (2012). *J. Appl. Cryst.* **45**, 849–854.

[bb5] Groom, C. R. & Allen, F. H. (2014). *Angew. Chem. Int. Ed.* **53**, 662–671. 10.1002/anie.201306438 24382699

[bb6] Kennard, C. H. L., Smith, G. & White, A. H. (1982). *Acta Cryst.* B**38**, 868–875.

[bb7] Liu, H.-L., Guo, S.-H., Li, Y.-Y. & Jian, F.-F. (2009). *Acta Cryst.* E**65**, o1905. 10.1107/S1600536809026919 PMC2977423 21583595

[bb8] Odendal, J. A., Bruce, J. C., Koch, K. R. & Haynes, D. A. (2010). *CrystEngComm*, **12**, 2398–2408.

[bb9] Pernak, J., Syguda, A., Janiszewska, D., Materna, K. & Praczyk, T. (2011). *Tetrahedron*, **67**, 4838–4844.

[bb10] Sheldrick, G. M. (2008). *Acta Cryst.* A**64**, 112–122. 10.1107/S0108767307043930 18156677

[bb11] Sieron, L., Kobylecka, J. & Turek, A. (2011). *Organic Chemistry International*, Volume 2011, Article ID 608165, 5 pages. 10.1155/2011/608165.

[bb12] Smith, G. (2014). *Acta Cryst.* C**70**, 315–319. 10.1107/S2053229614002459 24594725

[bb13] Smith, G. & Kennard, C. H. L. (1981). *Cryst. Struct. Commun.* **10**, 295–299.

[bb14] Smith, G., Lynch, D. E., Sagatys, D. S., Kennard, C. H. L. & Katekar, G. F. (1992). *Aust. J. Chem.* **45**, 1101–1108.

[bb15] Spek, A. L. (2009). *Acta Cryst.* D**65**, 148–155. 10.1107/S090744490804362X PMC2631630 19171970

[bb16] Zumdahl, R. L. (2010). In *A History of Weed Science in the United States*. New York: Elsevier.

